# Genetically predicted asthma and the risk of abnormal spermatozoa

**DOI:** 10.3389/fgene.2024.1377770

**Published:** 2024-05-23

**Authors:** Zhichao Li, Zhihai Teng, Zhenwei Han, Yanping Zhang, Yaxuan Wang

**Affiliations:** Department of Urology, The Second Hospital of Hebei Medical University, Shijiazhuang, China

**Keywords:** asthma, causality, abnormal spermatozoa, male infertility, mendelian randomization

## Abstract

**Background:**

Several previous animal and human studies have found a strong association between asthma and spermatozoa quality, but whether these associations are causal or due to bias remains to be elucidated.

**Methods:**

We performed a two-sample Mendelian randomization (MR) analysis to assess the causal effect of genetically predicted asthma on the risk of abnormal spermatozoa. Asthma, childhood-onset asthma (COA), and adult-onset asthma (AOA) (sample sizes ranging from 327,670 to 408,442) were included as the exposures. Genetic information for abnormal spermatozoa was obtained from a genome-wide association study (GWAS) comprising 209,921 participants. In univariable MR (UVMR) analysis, the inverse variance weighted (IVW) method was conducted as the primary method, with the MR Egger and weighted median used as supplementary methods for causal inference. Sensitivity analyses, including the Cochran Q test, Egger intercept test, MR-PRESSO, and leave-one-out analysis, were performed to verify the robustness of the MR results. Multivariable MR (MVMR) was conducted to evaluate the direct causal effects of asthma on abnormal spermatozoa risk.

**Results:**

UVMR detected causal associations between genetically predicted asthma and an increased risk of abnormal spermatozoa (OR: 1.270, 95% CI: 1.045–1.545, *p* = 0.017). Moreover, we found that AOA (OR: 1.46, 95% CI: 1.051, 2.018, *p* = 0.024) has positive causal effects on the risk of abnormal spermatozoa rather than COA (*p* = 0.558). Sensitivity analysis found little evidence of bias in the current study (*p* > 0.05). MVMR further confirmed that asthma directly affected the risk of abnormal spermatozoa.

**Conclusion:**

Our MR study suggested that genetically predicted asthma could be associated with an increased risk of abnormal spermatozoa, and similar results were obtained in AOA. Further studies are warranted to explain the underlying mechanisms of this association and may provide new avenues for prevention and treatment.

## 1 Introduction

As a worldwide human health problem, infertility is estimated to affect 8%–12% of couples globally. Male infertility contributes to more than half of all cases of global childlessness (Agarwal et al., 2021). It is noteworthy that the rate of male infertility is still on the rise and may have a severe negative impact on social development (Vollset et al., 2020). To date, the cause of male infertility has not been fully elucidated yet. Still, most of the time, it can be related to overweight, diabetes (Service et al., 2023), and poor lifestyle habits such as smoking (Sharma et al., 2016) and alcohol consumption (Basic et al., 2023), which may impair spermatogenesis and eventually lead to the production of abnormal spermatozoa. Moreover, It was reported that more than 2% of infertile men exhibit abnormal spermatozoa (Kumar and Singh, 2015). Asthma is one of the most common chronic inflammatory diseases, which has affected nearly 334 million patients worldwide at any age (Papi et al., 2018). It is characterized by a range of respiratory symptoms and airflow limitation to varied degrees, which can be generated by a range of airway inflammation and bronchospasm (Papi et al., 2018). As illustrated in animal models, male mice with asthma were related to abnormal spermatozoa and testicular structure (Xu et al., 2016; Deng et al., 2021; Wang et al., 2021; Feng et al., 2022). Moreover, a cross-sectional study in humans showed that men with asthma had lower spermatozoa concentration and total spermatozoa count compared with non-asthmatic men (Pedersen et al., 2023). The findings from the above studies have proved a negative correlation between asthma and spermatozoa quality. However, the underlying mechanisms linking asthma to spermatozoa quality are poorly understood. As reported, asthma can induce systemic inflammation and immune responses, leading to elevated levels of pro-inflammatory factors in the peripheral blood and excessive production of reactive oxygen species (Papi et al., 2018). These factors can adversely affect reproductive cells and interfere with spermatozoa production and maturation (Hasan et al., 2022).

Due to variances in the exposure to risk factors such as smoking, obesity, and alcohol consumption between humans and animals, the reliability of animal studies needs to be further investigated, and the conclusions may be biased. Additionally, considering the scarcity of relevant human studies, the presence of reverse causation, and the impact of confounding factors, it is exceedingly challenging to determine causality based on observational studies. Mendelian randomization (MR), utilizing genetic associations to investigate the causal impact of a risk factor on an outcome (Smith and Ebrahim, 2003), is a powerful and effective method for gauging causal inference. This approach can limit reverse causality and significantly reduce the likelihood of residual confounding (Davies et al., 2018). Therefore, we employed MR to circumvent limitations observed in previous research endeavors and to assess potential genetic causal associations between genetically predicted asthma and the risk of abnormal spermatozoa, intending to provide some insights into future prevention and treatment strategies.

## 2 Methods

### 2.1 Study design

Independent single nucleotide polymorphisms (SNPs) from genome-wide association studies (GWAS) were selected as instrumental variables (IVs). MR relies on three fundamental assumptions to ensure the reliability of results (Smith and Ebrahim, 2003): a close association between the IVs and exposure; IVs be independent of confounding factors of outcome; IVs can affect outcome only through exposure and not other means.

**FIGURE 1:**
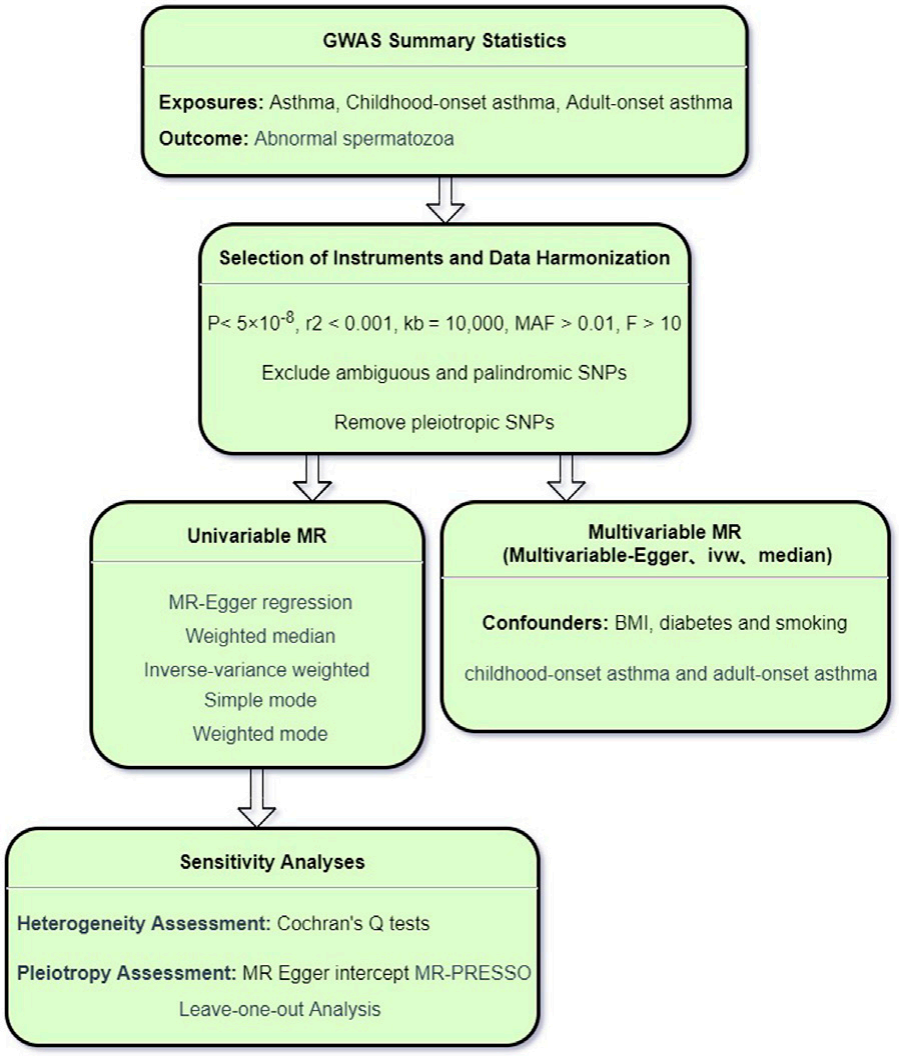
The schematic diagram of entire process.

In this study, asthma and abnormal spermatozoa were designated as the exposure and outcome, respectively. Initially, we employed the univariable MR (UVMR) approach to investigate the causal relationship between asthma and abnormal spermatozoa. Subsequently, we delved into the age-specific causal associations between asthma and abnormal spermatozoa. Following this, the multivariable MR (MVMR) was applied to further assess the direct impact of asthma on abnormal spermatozoa, adjusting for potential confounding factors such as obesity, diabetes (Service et al., 2023), and smoking (Sharma et al., 2016). Additionally, MVMR was utilized to evaluate age-specific causal relationships for further validation. The entire process is illustrated in the schematic diagram presented in Figure 1. The MR Study is based on publicly available GWAS data. Therefore, there is no need to seek patient consent and ethics committee approval.

### 2.2 Data sources

#### 2.2.1 Data sources of asthma and risk factors

The summary statistics of asthma were from the latest large-scale GWAS meta-analysis of 408,442 Europeans (56,167 cases and 352,255 controls) from the UK Biobank (Valette et al., 2021). These GWASs adjusted for age, sex, and the first 20 ancestry-based principal components. Furthermore, for childhood-onset asthma (COA) and adult-onset asthma (AOA), the summary data were extracted from the UK Biobank, including 356,083 people of European ancestry with self-reported physician-diagnosed asthma (Pividori et al., 2019). COA is defined as the age of asthma onset ≤12 years old (including 9,433 cases (age 6 ± 3 years, 59.3% men) and 318,237 controls). In comparison, AOA is defined as the age of asthma onset ≥26 years old (including 21,564 cases (age 44 ± 10 years, 36.4% men) and 318,237 controls, excluding individuals with chronic obstructive pulmonary disease, emphysema, or chronic bronchitis) (Pividori et al., 2019).

The GWAS summary data for the traditional risk factors of abnormal spermatozoa, including type 2 diabetes, body mass index, and Current tobacco smoking, were obtained from the corresponding consortia (Bycroft et al., 2018; Barton et al., 2021; Sakaue et al., 2021).

#### 2.2.2 Data sources of abnormal spermatozoa

The FinnGen study is a large (*n* = 218,792 in round 5) population biobank based in Finland, described in detail elsewhere (FinnGen, 2021; Kurki et al., 2023). GWAS summary data on the FinnGen cohort (round 5) includes a total of 915 individuals with abnormal spermatozoa and 209,006 country-matched participants of the European population (FinnGen phenotype ID: R18 ABNORMAL SPERMATOZ). These GWASs exclude individuals with azoospermia and abnormal findings upon examination of various body fluids, substances, and tissues, such as bronchial washings, nasal secretions, pleural fluid, sputum, peritoneal fluid, saliva, etc.

### 2.3 Selection of genetic instrument

To ensure the validity of the IVs, we conducted a screening process to select eligible SNPs that satisfied the three MR assumptions. First, to identify SNPs strongly correlated with exposure, we implemented a rigorous screening with a significant level of *p* < 5 × 10^−8^. Secondly, to identify the independent SNPs, these SNPs with the linkage disequilibrium (LD) need to be removed. The clumping process (r^2^ = 0.001, kb = 10,000) was conducted to assess the LD. For SNPs absent in the outcome, proxies were identified in high LD (r^2^ > 0.8) based on the European reference panel of the 1000 Genomes Project. We discarded those that were absent and had no appropriate proxies identified. Harmonization was then conducted to align the allele of the SNPs on outcome and exposure and discard palindromic SNPs with intermediate minor allele frequency (MAF > 0.42) and SNPs with incompatible alleles. Moreover, the F-statistics of selected SNPs should be above 10 to avoid weak instrument bias (Burgess et al., 2011). The F-statistics were calculated using the formula $F=R^{2}*\left(N-2\right)/\left(1-R^{2}\right)$ (Palmer et al., 2012; Levin et al., 2020). The N, and R^2^ of the formula represent the sample size, and the variance interpreted by the IVs, respectively. We estimated the proportion of variance in the phenotype explained by each SNP (phenotypic variance explained, PVE) using this equation (Shim et al., 2015): $\text{PVE}=\frac{2\times \left({beta}^{2})\times MAF\times (1-MAF\right)}{2\times \left({beta}^{2}\right)\times MAF\times \left(1-MAF\right)-{\left(SE\left(beta\right)\right)}^{2}\times N\times MAF\times \left(1-MAF\right)}$ . Where, N represents the sample size of the panel, beta is the effect for the genetic variant (SNP) of interest, SE (beta) is the standard error of effect for the genetic variant (SNP) of interest, MAF is the minor allele frequency for the genetic variant (SNP) of interest. Finally, we also excluded SNPs associated with the outcome and potential confounders by using the GWAS Catalog databases (https://www.ebi.ac.uk/gwas/) (Sollis et al., 2023).

All GWAS used in our MR analysis were exclusively limited to European ancestry to minimize potential bias from population heterogeneity. In addition, there was no overlap in GWAS data for exposure and outcomes, ensuring the independence of the datasets and enhancing the credibility of our MR analysis.

### 2.4 Statistical analysis

#### 2.4.1 UVMR and MVMR analysis

For UVMR, the inverse variance weighted (IVW) method served as the primary approach to calculate causal estimates in our analysis. Moreover, other MR analyses, like MR-Egger regression, weighted median (WM), simple mode, and weighted mode, were implemented to supplement IVW and provided more reliable estimates in a wider range of situations. The IVW method uses a meta-analytical approach to combine the wald ratios for each SNP, and it provides the most precise estimates when all IVs are valid (Burgess et al., 2016). The WM method and MR-Egger regression are more robust to the inclusion of instrumental variants with potential pleiotropy, which may, nevertheless, have lower precision and statistical power. The WM method allows for 50% of the instrumental variables to be invalid (Hartwig et al., 2017), and the MR-Egger method provides an intercept to indicate average pleiotropic bias (Bowden et al., 2015). As an additional step, we used weighted mode and simple mode to enhance accuracy and stability (Zhang et al., 2023). Additionally, we also performed the MR Steiger directionality test to infer causal direction (Hemani et al., 2017).

To determine whether the observed significant effect was a direct or indirect impact, we further conducted MVMR (Burgess and Thompson, 2015) with adjustment for the traditional risk factors of the abnormal spermatozoa, such as diabetes, BMI, and smoking. Similarly, IVW, WM, and MR-Egger regression were used for analysis, and the intercept derived from MR-Egger regression was used to detect potential horizontal pleiotropy. In addition, we applied MVMR to validate further the causal relationship between asthma and the risk of abnormal spermatozoa at different ages.

#### 2.4.2 Sensitivity analyses

Various sensitivity analyses were conducted to evaluate the strength of the results, which contained the heterogeneity and pleiotropy of IVs. Cochran’s Q tests of IVW and the MR-Egger approach were used to assess the IV heterogeneity, with a *p*-value of >0.05 indicating the lack of heterogeneity (Burgess S, 2021). The MR Egger intercept and MR-PRESSO test were also further conducted to identify the potential horizontal pleiotropic effects of enrolled IVs(Hemani et al., 2018; Verbanck et al., 2018). The leave-one-out sensitivity method was used to assess if one SNP significantly influenced causality estimates (Hemani et al., 2017). Furthermore, scatter diagrams were employed to evaluate the influence of anomalies on the findings, whereas funnel diagrams were used to assess the reliability of the correlation and the lack of variability.

For the evidence of significant causal effects, all the *p*-values were set at <0.05. All the statistical analyses were conducted using the publicly available R computational environment (version 4.2.3). The R packages “TwoSampleMR”, “MRPRESSO,” and “MendelianRandomization” were used for MR analysis and sensitivity analysis, respectively.

## 3 Results

### 3.1 Screening of genetic tools

We employed a genome-wide significance threshold (*p* < 5 × 10^−8^, r^2^ < 0.001, kb = 10,000) to identify GWAS-significant SNPs. The number of instrumental variables and the explained variances are shown in Table 1. All the SNPs included in the study had F-statistic greater than 10 (range: 29–512), indicating that the IVs were strong instruments and thus reducing the bias of IVs estimates. All the SNPs used in this study and their F-statistic are presented in Supplementary Table S1 in the Supplementary Document.

**TABLE 1:** Summary of the genome-wide association studies.

Exposure	NSNP	Sample	R2 (%)	F	Population	PMID/GWASID
Asthma	76	408,442	1.34	69.42	European	34,103,634
COA	45	327,670	1.07	77.31	European	31,036,433
AOA	16	339,801	0.25	53.99	European	31,036,433
Type 2 diabetes	145	490,089	-	-	European	34,594,039
Body mass index	309	457,756	-	-	European	34,226,706
Current tobacco smoking	32	462,434	-	-	European	ukb-b-223

### 3.2 The results of UVMR analysis

**FIGURE 2:**
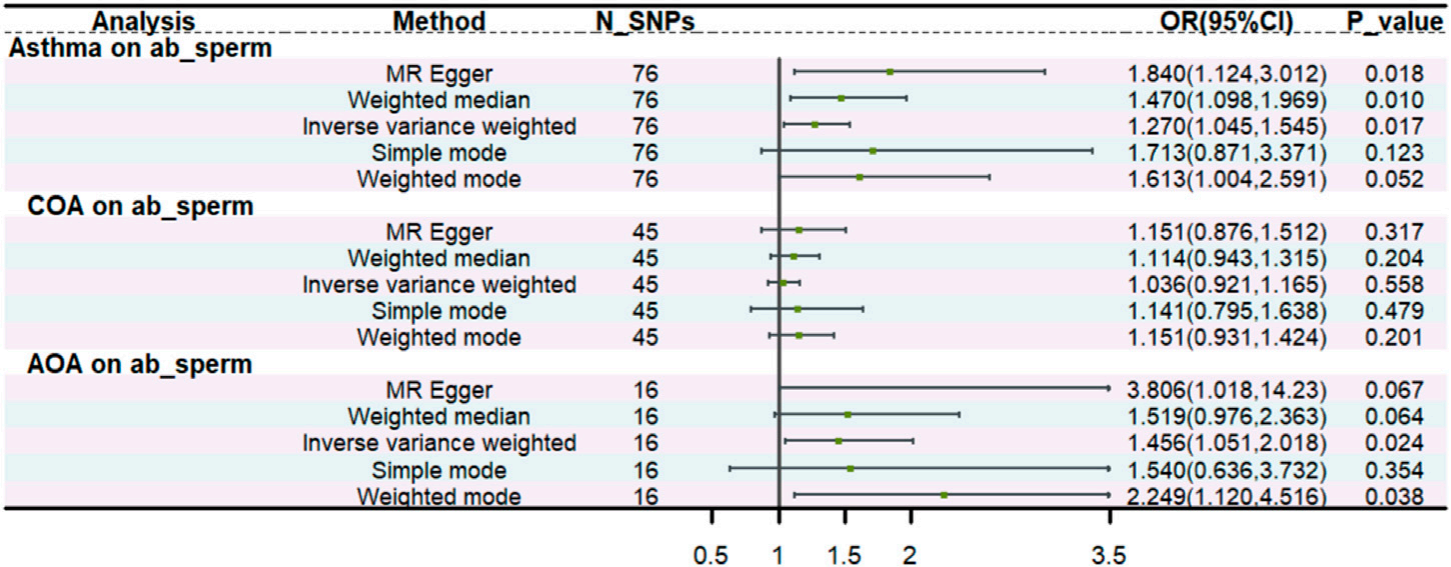
The univariable MR.

We found a statistically significant causal relationship between genetically predicted asthma and an increased risk of abnormal spermatozoa (OR: 1.270, 95% CI: 1.045–1.545, *p* = 0.017). Moreover, consistent results were shown by MR-Egger (OR:1.84, 95% CI: 1.124,3.012, *p* = 0.018), weighted median (OR:1.47, 95% CI: 1.098,1.969, *p* = 0.010). Additionally, we found AOA was associated with an increased risk of abnormal spermatozoa (OR:1.46, 95% CI: 1.051,2.018, *p* = 0.024). The causal inference was further supported by consistent direction and magnitude from distinct MR models. Nevertheless, no causal effect was found between COA and abnormal spermatozoa (*p* = 0.558). All the details are presented in Figure 2 (Supplementary Figures). The MR-Steiger test showed that the SNPs explained more variance in exposure than the outcome, which identified the robustness of the causal effect estimates.

### 3.3 Sensitivity tests

The *p*-values of the Q statistics calculated in the Cochran Q test of IVW and MR-Egger are much greater than 0.05. hence, there was no heterogeneity between these IVs. The MR-Egger intercept showed no statistical difference from the origin point (*p* > 0.05), suggesting no horizontal pleiotropy. MR-PRESSO global test also showed that no potential outlier could affect our estimation substantially (Table 2). Leave-one-out analysis indicated that the causalities of the positive results were very robust (Supplementary Figures).

**TABLE 2:** Sensitivity tests.

Exposure	Methods	Heterogeneity		Pleiotropy		Global test
		Q	*p*-Value	Intercept	*p*-value	*p*-value
Asthma	IVW	76.107	0.443			
	MR-Egger	73.535	0.493	−0.027	0.113	
	MR-PRESSO					0.456
COA	IVW	53.712	0.15			
	MR-Egger	52.84	0.145	−0.018	0.404	
	MR-PRESSO					0.145
AOA	IVW	15.09	0.445			
	MR-Egger	12.92	0.533	−0.083	0.163	
	MR-PRESSO					0.417

### 3.4 The results of MVMR analysis

**FIGURE 3:**
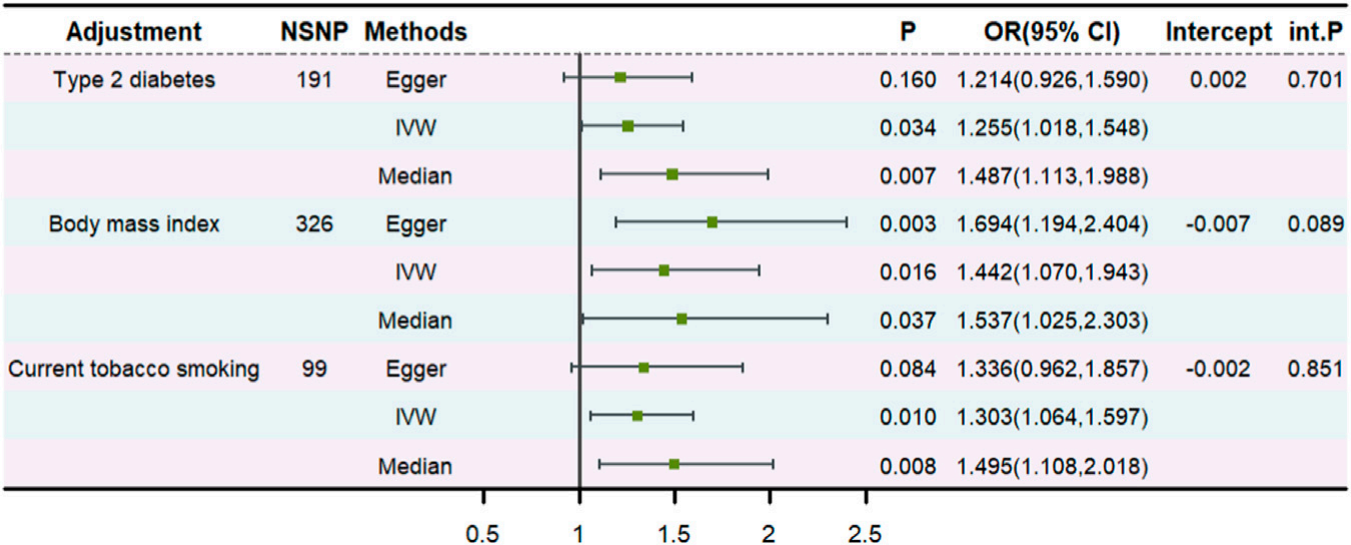
The multivariable MR.

To determine whether asthma exerted an impact on the risk of abnormal spermatozoa directly, we further conducted an MVMR analysis. The effect of genetically predicted asthma on abnormal spermatozoa remained after accounting for diabetes, BMI, and smoking (Figure 3). The intercept term derived from MR-Egger did not detect potential horizontal pleiotropy. Additionally, adjusting for COA, a suggestive positive association between genetically predicted AOA and abnormal spermatozoa remained (OR: 1.525, 95% CI: 0.977–2.379, *p* = 0.063) (Supplementary Table S2).

## 4 Discussion

To the best of our knowledge, this is the first study to evaluate the potential causal associations of asthma with abnormal spermatozoa based on the MR. This MR study consistently suggests that genetically predicted asthma was associated with an increased risk of abnormal spermatozoa. Furthermore, The causal inference was further supported by consistent direction and magnitude from distinct MR models. Moreover, we found that AOA has positive causal effects on the risk of abnormal spermatozoa rather than COA.

Our finding on the association between asthma and abnormal spermatozoa risk is consistent with previous studies. Recently, Multiple mouse model studies consistently indicate an association between asthma and spermatozoa quality, primarily characterized by significantly lower spermatozoa concentration and motility in the asthma mouse group compared to the control group (Xu et al., 2016; Deng et al., 2021; Wang et al., 2021; Feng et al., 2022). Xu et al. (2016) propose that asthma could activate the mitochondrial apoptosis signaling pathway in the mouse testis, thereby impacting spermatozoa quality. Similarly, Feng et al. (2022) argue that asthma can modulate the HIF-1 signaling pathway, resulting in increased expression of IL6, Stat3, HIF-1α protein, and mRNA, ultimately leading to spermatozoa apoptosis. Additionally, Deng et al. (2021) suggest that asthma can induce the production of abnormal spermatozoa by upregulating the expression of IL-6, vascular endothelial growth factor A, and mRNA while inhibiting albumin expression levels. In addition, only one cross-sectional study in humans found a potential association between self-reported asthma patients and reduced testicular function, primarily manifested by decreased spermatozoa concentration and total spermatozoa count. However, no significant differences were observed in semen volume, spermatozoa motility, or spermatozoa morphology (Pedersen et al., 2023). The findings from the above studies have proved a negative correlation between asthma and spermatozoa quality.

Although the underlying mechanisms of these associations are poorly understood, there are still some potential mechanisms that can be used to explain their possible connection. Asthma is characterized by reversible airflow obstruction, bronchial hyperresponsiveness, and airway inflammation. Th17 cells, a subset of CD4^+^ T cells, have been associated with asthma phenotypes (Newcomb and Peebles, 2013). These cells produce IL-17, driven by TGF-β, IL-1β, IL-6 and IL-23. In the synergistic action of TNF-α, IL-17 stimulates the secretion of IL-1β, IL-6, and GM-CSF by airway epithelial cells, endothelial cells, and fibroblasts, leading to neutrophil recruitment ultimately (Alcorn et al., 2010). Additionally, IFN-γ, a pleiotropic Th1 cytokine, also plays a crucial role in asthma pathogenesis (Raundhal et al., 2015). The pro-inflammatory state is activated in response to invading pathogens, and a significant amount of pro-inflammatory cytokines, such as IL-1, IL-6, and TNF-α, are secreted by Th1 cells, CD4^+^ T cells, macrophages, and dendritic cells (Turner et al., 2014). These cytokines stimulate, recruit, and amplify immune cells, driving an inflammatory reaction. In addition to their role in the pathogenesis of asthma, IFN-γ, IL-6, TNF-α, and other cytokines have also been implicated in the development of decreased spermatozoa quality. Studies have demonstrated that seminal plasma from men with poor spermatozoa motility had higher concentrations of IFN-γ, TNF-α, and IL-10 (Ulcova-Gallova et al., 2009). Similarly, IFNγ, IL-17, and IL-1β can impair spermatozoa motility and viability (Paira et al., 2022). Several previous studies have shown that pro-inflammatory cytokines such as IL-6, IFN-γ, IL-1β, and TNF-α can induce the overproduction of reactive oxygen species, leading to the formation of oxidative stress. This negatively affects the process of spermatogenesis and maturation ultimately (Lampiao and du Plessis, 2008; Dobrakowski et al., 2017; Aitken et al., 2022). There is a very complex relationship between asthma and sperm homeostasis. Recent studies have indicated that the genetic structures of COA and AOA are somewhat distinct. Interestingly, variants that are linked to obesity and smoking appear to have a more significant contribution to the risk of AOA than COA (Ferreira et al., 2019). Additionally, several studies (Toskala and Kennedy, 2015; Agarwal et al., 2021) suggest that asthma and spermatozoa quality are influenced by various common factors, such as diabetes, obesity, and smoking. However, the MVMR study confirmed that the effect of genetically predicted asthma on abnormal spermatozoa remained after accounting for diabetes, BMI, and smoking.

In summary, It appears that asthma may increase the risk of abnormal spermatozoa through the mechanism of inflammatory processes and oxidative stress. Future advancements should focus on further research and conducting large-scale prospective human studies to elucidate their underlying mechanisms and identify potential therapeutic targets.

The major strength of this study is the MR method, which is less susceptible to potential confounding factors and other biases, thus reinforcing the causal inference. Second, we assessed the potential associations using summary statistics from several large GWAS. In addition, the results remained overall consistent across several sensitivity analyses. Moreover, our findings were unlikely to be impacted by population structure bias since the analyses were restricted to individuals of European ancestry. Limitations need to be considered in this study. First, the nonlinear connection between asthma and the risk of abnormal spermatozoa cannot be eliminated due to the linear effect assumption in MR analysis. Furthermore, our study is mainly based on Europeans. Thus, the generalization of the findings to other ethnic groups needs to be cautious. Furthermore, due to the absence of suitable GWAS data, we refrained from conducting further analysis on various subtypes of asthma. Finally, there are other possible unmeasured and residual confounding factors, like environmental factors, asthma medications, allergic diseases, or other pulmonary conditions, which might drive the bias of the overall estimates.

## 5 Conclusion

To summarize, the MR study provides evidence supporting a causal relationship between genetically determined asthma and the increased risk of abnormal spermatozoa, and similar results are obtained in AOA. Our research findings hold significant implications for public health and epidemiological prevention. Clinical practice should emphasize early prevention and preconception counseling. Additionally, we should promote multidisciplinary collaboration to formulate comprehensive guidelines, strengthening reproductive health management for asthmatic men of childbearing age, aiming to achieve early detection and timely prevention.

## Data Availability

The datasets presented in this study can be found in online repositories. The names of the repository/repositories and accession number(s) can be found in the article/Supplementary Material.
