# Supplementary material for: Genetically predicted asthma and the risk of abnormal spermatozoa
Source: Front Genet. 2024 May 23;15:1377770. doi: 10.3389/fgene.2024.1377770 (PMC11153665; doi:10.3389/fgene.2024.1377770)
Supplement: Supplementary file 1 [file DataSheet1.docx]

Supplementary Material

# Supplementary Figures and Tables

## Supplementary Tables

## Table S1. The parameter of all snps in this study

| exposure | SNP | A1 | A2 | chr | beta | eaf | se | pval | F |
| --- | --- | --- | --- | --- | --- | --- | --- | --- | --- |
| Asthma | rs10178845 | A | G | 2 | -0.06166 | 0.294552 | 0.007172 | 8.22E-18 | 74 |
| Asthma | rs10477741 | G | T | 5 | 0.074266 | 0.128434 | 0.009797 | 3.44E-14 | 57 |
| Asthma | rs10486391 | G | A | 7 | -0.03886 | 0.410809 | 0.00665 | 5.13E-09 | 34 |
| Asthma | rs10912564 | T | C | 1 | 0.039258 | 0.306507 | 0.00711 | 3.37E-08 | 30 |
| Asthma | rs11042902 | T | C | 11 | 0.041331 | 0.307493 | 0.007111 | 6.17E-09 | 34 |
| Asthma | rs11071559 | T | C | 15 | -0.08469 | 0.128023 | 0.009765 | 4.21E-18 | 75 |
| Asthma | rs11088309 | G | C | 21 | 0.060458 | 0.142544 | 0.009315 | 8.55E-11 | 42 |
| Asthma | rs11178649 | T | G | 12 | -0.04293 | 0.407176 | 0.006647 | 1.06E-10 | 42 |
| Asthma | rs112267124 | A | G | 17 | 0.042884 | 0.234223 | 0.007699 | 2.55E-08 | 31 |
| Asthma | rs113981909 | A | G | 10 | -0.0614 | 0.115023 | 0.010526 | 5.45E-09 | 34 |
| Asthma | rs11567923 | CT | C | 10 | 0.04979 | 0.193178 | 0.00839 | 2.95E-09 | 35 |
| Asthma | rs117552144 | T | C | 19 | 0.079499 | 0.066752 | 0.013807 | 8.51E-09 | 33 |
| Asthma | rs117710327 | A | C | 19 | -0.12641 | 0.066958 | 0.013395 | 3.84E-21 | 89 |
| Asthma | rs11816044 | A | G | 10 | -0.04733 | 0.325637 | 0.006981 | 1.20E-11 | 46 |
| Asthma | rs12123821 | T | C | 1 | 0.145713 | 0.048173 | 0.015313 | 1.81E-21 | 91 |
| Asthma | rs12165508 | C | T | 22 | -0.04514 | 0.795068 | 0.008092 | 2.42E-08 | 31 |
| Asthma | rs12365699 | A | G | 11 | -0.05354 | 0.166687 | 0.008796 | 1.16E-09 | 37 |
| Asthma | rs12964116 | G | A | 18 | 0.107619 | 0.035462 | 0.017652 | 1.08E-09 | 37 |
| Asthma | rs13277355 | G | A | 8 | -0.04056 | 0.725959 | 0.007326 | 3.09E-08 | 31 |
| Asthma | rs1444782 | A | G | 10 | -0.09545 | 0.423577 | 0.0066 | 2.14E-47 | 209 |
| Asthma | rs148639908 | AT | A | 6 | -0.07073 | 0.353557 | 0.00682 | 3.35E-25 | 108 |
| Asthma | rs1608555 | T | C | 7 | 0.037747 | 0.358532 | 0.006822 | 3.14E-08 | 31 |
| Asthma | rs1684466 | A | G | 3 | -0.05689 | 0.637709 | 0.007012 | 4.92E-16 | 66 |
| Asthma | rs1689510 | C | G | 12 | 0.055648 | 0.340371 | 0.006881 | 6.09E-16 | 65 |
| Asthma | rs16903574 | G | C | 5 | 0.086551 | 0.076512 | 0.012549 | 5.31E-12 | 48 |
| Asthma | rs17454584 | G | A | 4 | 0.059793 | 0.218612 | 0.007891 | 3.52E-14 | 57 |
| Asthma | rs174557 | G | A | 11 | -0.04547 | 0.309707 | 0.007055 | 1.15E-10 | 42 |
| Asthma | rs1837253 | C | T | 5 | 0.108356 | 0.739654 | 0.00748 | 1.49E-47 | 210 |
| Asthma | rs1870140 | G | A | 10 | -0.04979 | 0.845758 | 0.009066 | 3.96E-08 | 30 |
| Asthma | rs200491113 | GTAGA | G | 9 | -0.12878 | 0.032041 | 0.018736 | 6.28E-12 | 47 |
| Asthma | rs2296618 | G | A | 1 | -0.06188 | 0.135293 | 0.009629 | 1.30E-10 | 41 |
| Asthma | rs2412099 | A | G | 17 | -0.0513 | 0.42139 | 0.006627 | 9.89E-15 | 60 |
| Asthma | rs2477923 | C | T | 10 | -0.03613 | 0.463296 | 0.00656 | 3.64E-08 | 30 |
| Asthma | rs2800040 | A | G | 6 | -0.03752 | 0.406794 | 0.006849 | 4.29E-08 | 30 |
| Asthma | rs28498223 | T | C | 14 | 0.049732 | 0.280804 | 0.007315 | 1.05E-11 | 46 |
| Asthma | rs2988277 | T | C | 1 | -0.04412 | 0.398654 | 0.00666 | 3.49E-11 | 44 |
| Asthma | rs3024971 | G | T | 12 | -0.1122 | 0.107142 | 0.010627 | 4.67E-26 | 111 |
| Asthma | rs34290285 | A | G | 2 | -0.09887 | 0.255414 | 0.00751 | 1.38E-39 | 173 |
| Asthma | rs35225972 | A | G | 11 | 0.043814 | 0.682812 | 0.007023 | 4.42E-10 | 39 |
| Asthma | rs35320232 | T | C | 1 | -0.05578 | 0.118792 | 0.01013 | 3.67E-08 | 30 |
| Asthma | rs35441874 | A | T | 16 | -0.08247 | 0.247084 | 0.007615 | 2.47E-27 | 117 |
| Asthma | rs35467801 | GT | G | 5 | -0.04543 | 0.274803 | 0.007322 | 5.46E-10 | 39 |
| Asthma | rs35570272 | T | G | 3 | 0.050945 | 0.396277 | 0.006701 | 2.91E-14 | 58 |
| Asthma | rs35621564 | G | A | 7 | -0.04439 | 0.365249 | 0.006819 | 7.50E-11 | 42 |
| Asthma | rs368981 | A | G | 9 | 0.056184 | 0.708867 | 0.007227 | 7.57E-15 | 60 |
| Asthma | rs3785356 | T | C | 16 | 0.0563 | 0.29795 | 0.007163 | 3.85E-15 | 62 |
| Asthma | rs3827780 | A | G | 6 | -0.03685 | 0.555099 | 0.006567 | 2.02E-08 | 31 |
| Asthma | rs413214 | A | G | 5 | 0.046933 | 0.616158 | 0.006711 | 2.68E-12 | 49 |
| Asthma | rs4480384 | G | A | 1 | 0.043097 | 0.643257 | 0.006813 | 2.52E-10 | 40 |
| Asthma | rs4722758 | G | C | 7 | 0.063412 | 0.19958 | 0.00815 | 7.21E-15 | 61 |
| Asthma | rs4736639 | C | G | 8 | -0.03973 | 0.286228 | 0.007238 | 4.05E-08 | 30 |
| Asthma | rs4739738 | A | G | 8 | -0.06791 | 0.641572 | 0.006805 | 1.86E-23 | 100 |
| Asthma | rs4795401 | G | A | 17 | -0.10255 | 0.510615 | 0.006525 | 1.16E-55 | 247 |
| Asthma | rs479844 | G | A | 11 | 0.040322 | 0.554743 | 0.006556 | 7.70E-10 | 38 |
| Asthma | rs4842921 | A | G | 15 | -0.04071 | 0.387483 | 0.006691 | 1.17E-09 | 37 |
| Asthma | rs56375023 | A | G | 15 | 0.103455 | 0.237326 | 0.007693 | 3.18E-41 | 181 |
| Asthma | rs5743618 | A | C | 4 | -0.06644 | 0.225707 | 0.007861 | 2.86E-17 | 71 |
| Asthma | rs61816766 | C | T | 1 | 0.135284 | 0.033244 | 0.018687 | 4.51E-13 | 52 |
| Asthma | rs72823641 | A | T | 2 | -0.14784 | 0.13665 | 0.009618 | 2.56E-53 | 236 |
| Asthma | rs7423358 | C | T | 2 | 0.043039 | 0.758113 | 0.007648 | 1.83E-08 | 32 |
| Asthma | rs7626218 | T | A | 3 | -0.04135 | 0.395198 | 0.006679 | 5.96E-10 | 38 |
| Asthma | rs76493820 | G | C | 5 | 0.086374 | 0.057957 | 0.014066 | 8.21E-10 | 38 |
| Asthma | rs7734635 | G | A | 5 | 0.076781 | 0.154263 | 0.009066 | 2.48E-17 | 72 |
| Asthma | rs7770794 | A | G | 6 | 0.041555 | 0.285802 | 0.007224 | 8.81E-09 | 33 |
| Asthma | rs78556180 | T | G | 1 | 0.050681 | 0.146826 | 0.00923 | 3.99E-08 | 30 |
| Asthma | rs7936312 | T | G | 11 | 0.083833 | 0.476762 | 0.006528 | 9.54E-38 | 165 |
| Asthma | rs7961712 | A | G | 12 | 0.056485 | 0.849901 | 0.009172 | 7.35E-10 | 38 |
| Asthma | rs802731 | G | C | 6 | 0.047918 | 0.270854 | 0.007337 | 6.54E-11 | 43 |
| Asthma | rs848 | C | A | 5 | -0.09633 | 0.817872 | 0.008472 | 5.80E-30 | 129 |
| Asthma | rs912131 | G | A | 13 | 0.058077 | 0.703887 | 0.007137 | 4.04E-16 | 66 |
| Asthma | rs919826 | C | T | 9 | -0.03575 | 0.489954 | 0.006543 | 4.65E-08 | 30 |
| Asthma | rs9260752 | A | G | 6 | 0.180227 | 0.021188 | 0.032038 | 1.85E-08 | 32 |
| Asthma | rs9272226 | T | C | 6 | -0.08977 | 0.628207 | 0.009462 | 2.37E-21 | 90 |
| Asthma | rs9273386 | C | T | 6 | 0.133835 | 0.588625 | 0.009155 | 2.11E-48 | 214 |
| Asthma | rs981625 | G | C | 13 | 0.072473 | 0.06495 | 0.013216 | 4.16E-08 | 30 |
| Asthma | rs992969 | G | A | 9 | -0.11991 | 0.74764 | 0.007562 | 1.25E-56 | 251 |
| COA | rs10175070 | G | A | 2 | 0.115113 | 0.251 | 0.016816 | 4.31E-12 | 47 |
| COA | rs10414065 | C | T | 19 | 0.188138 | 0.934 | 0.032511 | 6.37E-09 | 33 |
| COA | rs10774625 | G | A | 12 | 0.083422 | 0.504 | 0.014782 | 2.07E-08 | 32 |
| COA | rs11071559 | C | T | 15 | 0.18648 | 0.872 | 0.023486 | 2.57E-15 | 63 |
| COA | rs11178648 | C | T | 12 | 0.083422 | 0.592 | 0.015252 | 4.16E-08 | 30 |
| COA | rs117137535 | A | G | 9 | 0.267734 | 0.026 | 0.04562 | 4.16E-09 | 34 |
| COA | rs12023876 | G | T | 1 | 0.097127 | 0.668 | 0.015971 | 1.11E-09 | 37 |
| COA | rs12365699 | G | A | 11 | 0.154436 | 0.833 | 0.020979 | 1.57E-13 | 54 |
| COA | rs12634152 | T | C | 3 | 0.124869 | 0.547 | 0.014865 | 1.08E-16 | 71 |
| COA | rs12964116 | G | A | 18 | 0.28968 | 0.036 | 0.035289 | 2.56E-16 | 67 |
| COA | rs1321859 | C | T | 6 | 0.097127 | 0.649 | 0.015746 | 9.32E-10 | 38 |
| COA | rs13277355 | A | G | 8 | 0.10436 | 0.274 | 0.016316 | 1.65E-10 | 41 |
| COA | rs13416555 | C | G | 2 | 0.122218 | 0.705 | 0.016478 | 1.94E-13 | 55 |
| COA | rs16903574 | G | C | 5 | 0.21188 | 0.077 | 0.026399 | 9.01E-16 | 64 |
| COA | rs1696361 | T | C | 12 | 0.103459 | 0.359 | 0.015186 | 1.19E-11 | 46 |
| COA | rs1837253 | C | T | 5 | 0.191446 | 0.74 | 0.017702 | 2.33E-27 | 117 |
| COA | rs1887704 | G | C | 13 | 0.116894 | 0.681 | 0.016332 | 5.48E-13 | 51 |
| COA | rs1950897 | C | T | 14 | 0.091667 | 0.287 | 0.016058 | 1.38E-08 | 33 |
| COA | rs2051809 | A | C | 5 | 0.161268 | 0.247 | 0.016716 | 3.24E-22 | 93 |
| COA | rs2066844 | T | C | 16 | 0.177309 | 0.048 | 0.032237 | 3.52E-08 | 30 |
| COA | rs2069763 | A | C | 4 | 0.118672 | 0.334 | 0.01541 | 1.59E-14 | 59 |
| COA | rs28407950 | C | T | 6 | 0.303063 | 0.756 | 0.01864 | 1.27E-59 | 264 |
| COA | rs3122929 | T | C | 12 | 0.125751 | 0.404 | 0.014851 | 6.59E-17 | 72 |
| COA | rs34290285 | G | A | 2 | 0.162969 | 0.745 | 0.017765 | 2.06E-20 | 84 |
| COA | rs34880821 | A | G | 7 | 0.10075 | 0.283 | 0.016151 | 4.73E-10 | 39 |
| COA | rs35032408 | T | G | 16 | 0.1415 | 0.785 | 0.018593 | 4.59E-14 | 58 |
| COA | rs35570272 | T | G | 3 | 0.09531 | 0.396 | 0.015072 | 2.68E-10 | 40 |
| COA | rs3785356 | T | C | 16 | 0.115113 | 0.297 | 0.015921 | 5.69E-13 | 52 |
| COA | rs4473914 | T | C | 7 | 0.088011 | 0.604 | 0.015182 | 9.02E-09 | 34 |
| COA | rs4574025 | T | C | 18 | 0.086178 | 0.535 | 0.014983 | 9.25E-09 | 33 |
| COA | rs4739738 | G | A | 8 | 0.110647 | 0.359 | 0.015299 | 3.45E-13 | 52 |
| COA | rs4795399 | T | C | 17 | 0.340749 | 0.529 | 0.015058 | 1.45E-111 | 512 |
| COA | rs479844 | G | A | 11 | 0.10075 | 0.555 | 0.01499 | 1.48E-11 | 45 |
| COA | rs4807630 | T | C | 19 | 0.088926 | 0.308 | 0.015862 | 2.06E-08 | 31 |
| COA | rs56062135 | T | C | 15 | 0.177309 | 0.237 | 0.016657 | 2.97E-26 | 113 |
| COA | rs5743618 | C | A | 4 | 0.222343 | 0.774 | 0.018799 | 3.19E-32 | 140 |
| COA | rs61894547 | T | C | 11 | 0.380489 | 0.052 | 0.028937 | 2.17E-39 | 173 |
| COA | rs705699 | A | G | 12 | 0.10075 | 0.425 | 0.01499 | 1.43E-11 | 45 |
| COA | rs72823641 | T | A | 2 | 0.34713 | 0.863 | 0.024331 | 3.01E-46 | 204 |
| COA | rs7518129 | G | A | 1 | 0.105261 | 0.31 | 0.015841 | 2.21E-11 | 44 |
| COA | rs7894791 | C | A | 10 | 0.098034 | 0.587 | 0.015255 | 9.43E-11 | 41 |
| COA | rs917115 | C | T | 7 | 0.10616 | 0.208 | 0.017885 | 1.73E-09 | 35 |
| COA | rs9391997 | G | A | 6 | 0.097127 | 0.527 | 0.015044 | 6.89E-11 | 42 |
| COA | rs943451 | T | C | 10 | 0.117783 | 0.318 | 0.015864 | 7.51E-14 | 55 |
| COA | rs992969 | A | G | 9 | 0.221542 | 0.252 | 0.016345 | 6.76E-42 | 184 |
| AOA | rs11088309 | G | C | 21 | 0.076035 | 0.143 | 0.013946 | 4.83E-08 | 30 |
| AOA | rs12617922 | G | A | 2 | 0.063913 | 0.518 | 0.010052 | 1.59E-10 | 40 |
| AOA | rs12788104 | G | A | 11 | 0.062035 | 0.688 | 0.011031 | 1.41E-08 | 32 |
| AOA | rs1321859 | C | T | 6 | 0.07325 | 0.649 | 0.010433 | 5.68E-12 | 49 |
| AOA | rs174621 | G | A | 11 | 0.068593 | 0.772 | 0.012144 | 1.46E-08 | 32 |
| AOA | rs17622378 | A | G | 5 | 0.074179 | 0.573 | 0.010191 | 3.17E-13 | 53 |
| AOA | rs1775554 | A | C | 10 | 0.114221 | 0.577 | 0.010246 | 5.17E-29 | 124 |
| AOA | rs1837253 | C | T | 5 | 0.084341 | 0.74 | 0.011486 | 2.77E-13 | 54 |
| AOA | rs28407950 | C | T | 6 | 0.138021 | 0.756 | 0.012002 | 7.67E-31 | 132 |
| AOA | rs34290285 | G | A | 2 | 0.077887 | 0.745 | 0.011801 | 2.16E-11 | 44 |
| AOA | rs35032408 | T | G | 16 | 0.070458 | 0.785 | 0.012354 | 1.10E-08 | 33 |
| AOA | rs56062135 | T | C | 15 | 0.079735 | 0.237 | 0.01178 | 6.27E-12 | 46 |
| AOA | rs56389811 | C | T | 12 | 0.075107 | 0.761 | 0.011823 | 2.36E-10 | 40 |
| AOA | rs72823641 | T | A | 2 | 0.103459 | 0.863 | 0.014949 | 3.72E-12 | 48 |
| AOA | rs7936312 | T | G | 11 | 0.058269 | 0.477 | 0.009864 | 4.10E-09 | 35 |
| AOA | rs992969 | A | G | 9 | 0.09531 | 0.252 | 0.01136 | 3.14E-17 | 70 |

## Table S2. Different statistical methods for multivariable MR

| Exposure | Adjustment | nSNP | Methods | pval |  | Intercept | int.P |
| --- | --- | --- | --- | --- | --- | --- | --- |
| Asthma | Type 2 diabetes | 145 | Egger | 0.16 | 1.214(0.926,1.590) | 0.002 | 0.701 |
|  |  |  | IVW | 0.034 | 1.255(1.018,1.548) |  |  |
|  |  |  | Median | 0.007 | 1.487(1.113,1.988) |  |  |
|  | Body mass index | 309 | Egger | 0.003 | 1.694(1.194,2.404) | -0.007 | 0.089 |
|  |  |  | IVW | 0.016 | 1.442(1.070,1.943) |  |  |
|  |  |  | Median | 0.037 | 1.537(1.025,2.303) |  |  |
|  | Current tobacco smoking | 32 | Egger | 0.084 | 1.336(0.962,1.857) | -0.002 | 0.851 |
|  |  |  | IVW | 0.01 | 1.303(1.064,1.597) |  |  |
|  |  |  | Median | 0.008 | 1.495(1.108,2.018) |  |  |
| AOA | COA | 44 | Egger | 0.089 | 1.590(0.932,2.716) | 0.025 | 0.835 |
|  |  |  | IVW | 0.063 | 1.525(0.977,2.380) |  |  |
|  |  |  | Median | 0.261 | 1.409(0.774,2.565) |  |  |

## Supplementary Figures

##
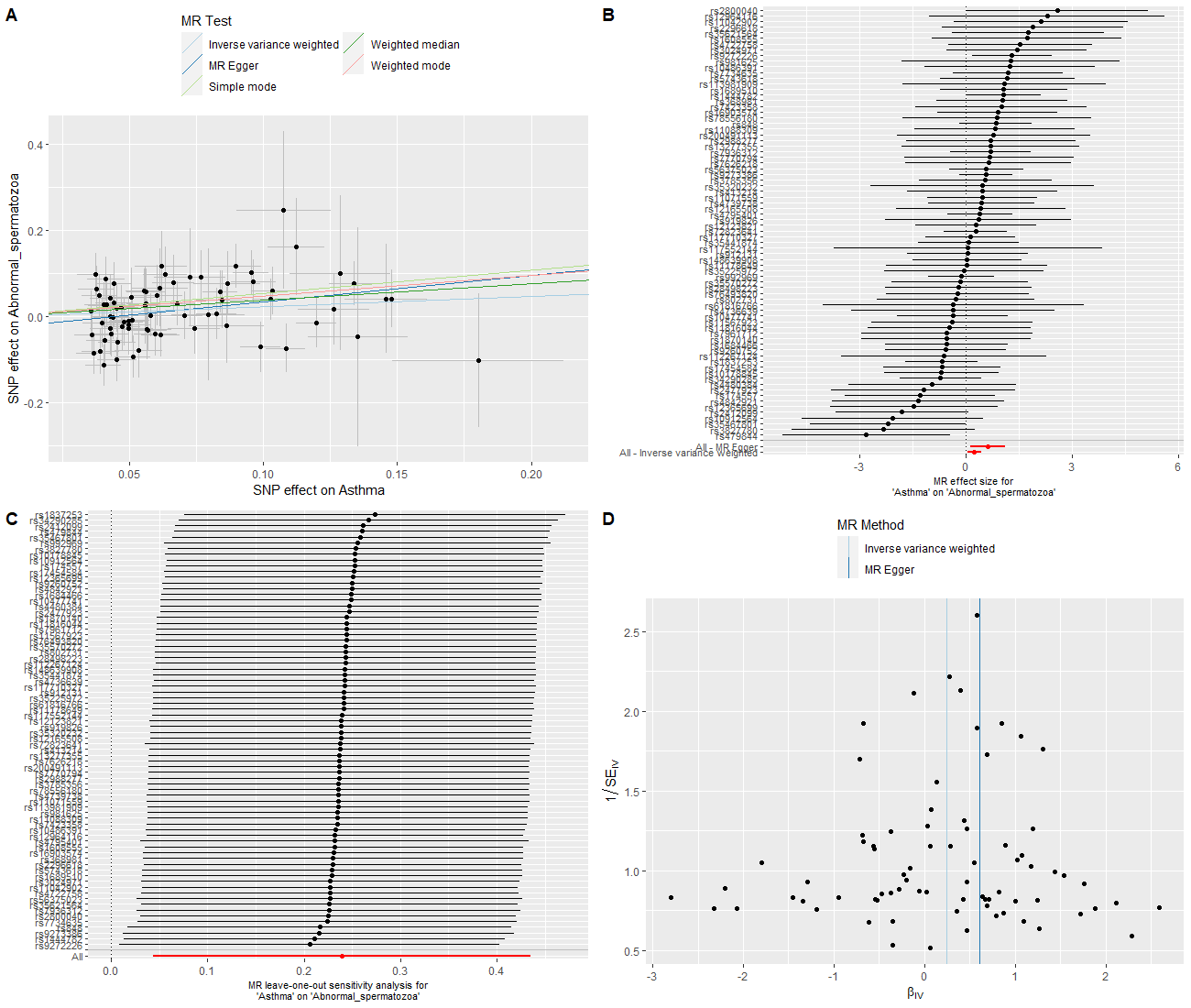


**Supplementary Figure 1.** Causal relationship between asthma and risk of abnormal spermatozoa (A) Scatter plot. (B) Forest plot. (C) Leave-one-out plot. (D) Funnel plot. OR, odds ratio; CI, confidence interval; IVW, inverse variance weighted; COA: childhood-onset asthma; AOA: adult-onset asthma.


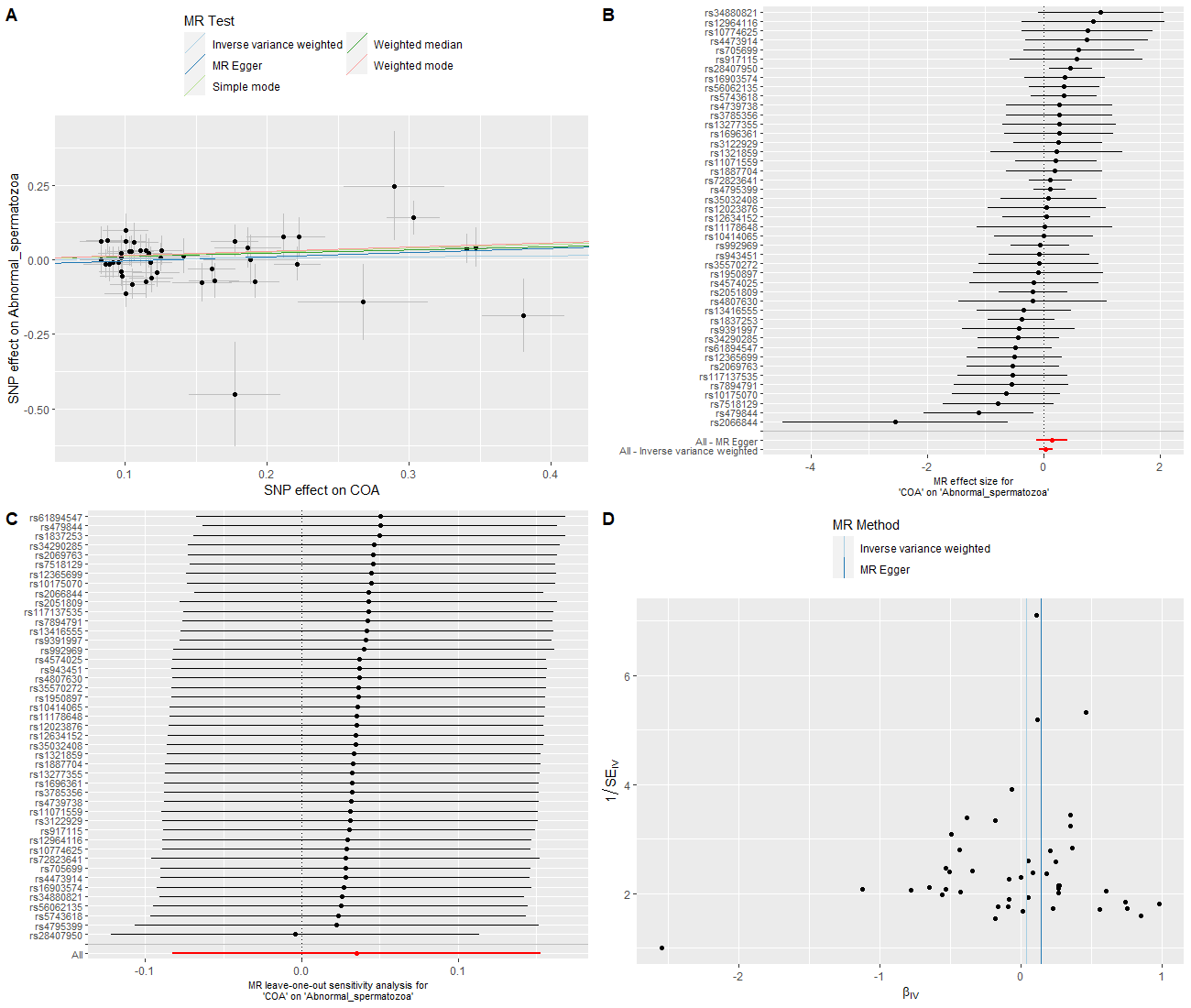


**Supplementary Figure 2.** Causal relationship between COA and risk of abnormal spermatozoa (A) Scatter plot. (B) Forest plot. (C) Leave-one-out plot. (D) Funnel plot. OR, odds ratio; CI, confidence interval; IVW, inverse variance weighted; COA: childhood-onset asthma; AOA: adult-onset asthma.


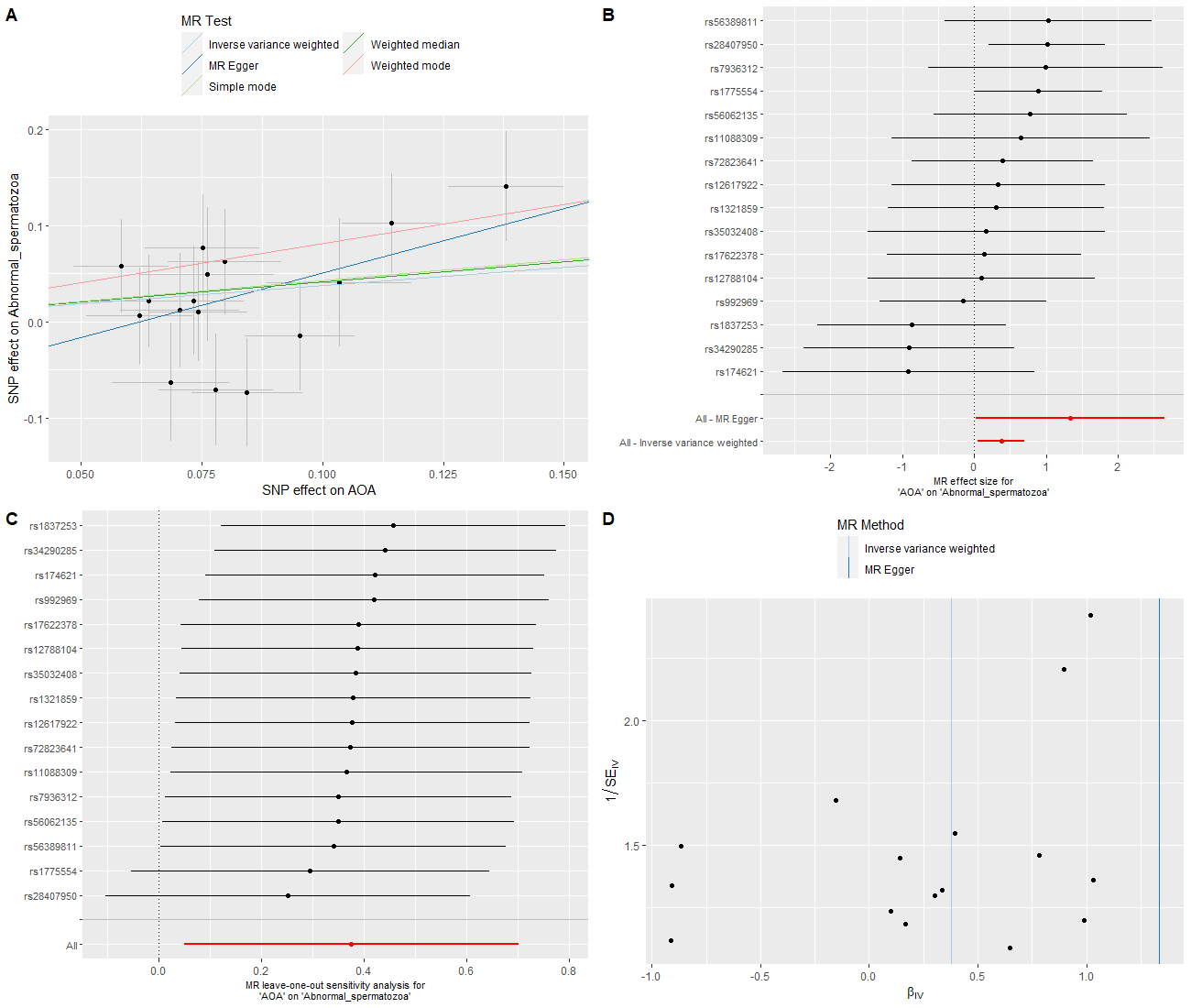


**Supplementary Figure 3.** Causal relationship between AOA and risk of abnormal spermatozoa (A) Scatter plot. (B) Forest plot. (C) Leave-one-out plot. (D) Funnel plot. OR, odds ratio; CI, confidence interval; IVW, inverse variance weighted; COA: childhood-onset asthma; AOA: adult-onset asthma.
